# Dopamine receptor loss of function is not protective of *rd1* rod photoreceptors in vivo

**Published:** 2009-12-23

**Authors:** Judith Mosinger Ogilvie, Angela M. Hakenewerth, Rachel R. Gardner, Joshua G. Martak, Virginia M. Maggio

**Affiliations:** Saint Louis University, Department of Biology, St. Louis, MO

## Abstract

**Purpose:**

The retinal degeneration (*rd1*) mouse undergoes a rapid loss of rod photoreceptors due to a defect in the cGMP-phosphodiesterase gene. We have previously demonstrated that dopamine (DA) antagonists or DA depletion blocks photoreceptor degeneration and that DA is necessary for photoreceptor degeneration in the *rd1* mouse retinal organ culture model. Antagonists for either D1- or D2-family DA receptors are protective in *rd1* organ cultures.

**Methods:**

To determine whether photoreceptor survival can be increased in vivo in the *rd1* mouse, we used both a pharmacological and a genetic approach. The pharmacological approach involved three techniques to administer 6-hydroxydopamine (6-OHDA) in an attempt to deplete DA in postnatal mouse retina in vivo. As a genetic alternative, DA receptor signaling was inactivated by crossbreeding *rd1* mice to D1, D2, D4, and D5 knockout mice to create four lines of double mutants.

**Results:**

Pharmacological DA depletion was incomplete due to the limiting size of the postnatal mouse eye and the lethality of systemic inhibition of DA signaling. In all four lines of double mutants, no increase in rod photoreceptor survival was observed. To determine whether protection of *rd1* photoreceptors by inhibition of dopaminergic signaling is a result of conditions specific to the organ culture environment, we grew in vitro retinas from the four lines of double mutant mice for four weeks. Again, no increase in photoreceptor survival was seen. Finally, three triple mutants were generated that lacked two DA receptors (D1/D2; D1/D4; and D2/D4) on a *rd1* background. In all three cases, rod photoreceptors were not protected from degeneration.

**Conclusions:**

The dramatic protection of *rd1* rod photoreceptors by inhibition of DA signaling in organ culture has not been reproduced in vivo by either a pharmacological approach, due to technical limitations, or by genetic manipulations. The possible role of compensatory effects during retinal development in DA receptor deficient mice is considered.

## Introduction

Retinitis pigmentosa (RP) is a genetically heterogeneous family of inherited degenerative diseases in the retina. In recent years, considerable progress has been made in elucidating the disease processes and their underlying molecular mechanisms, in large part due to availability of animal models of the disease. The *rd1* mouse is among the first identified [1] and best-characterized animal models of RP [2]. The defect is caused by a loss-of-function mutation in the β-subunit of the rod photoreceptor cGMP-phosphodiesterase gene (*PDE6b)* [3-5]. This results in rod photoreceptor cell death that begins by postnatal day 10 (P10) and is completed by P21, at which time only cone nuclei remain in the outer nuclear layer [6,7]. Mutations in *PDE6b* account for 4%–5% of human cases of RP [8-10], making the *rd1* mouse a particularly relevant model of human disease.

Numerous approaches are under study for treatment of photoreceptor degeneration—ranging from transplantation and prosthetic devices to stem cells, gene transfer, and pharmacological intervention using trophic factors or anti-apoptotic agents [11]. The *rd1* retinal organ culture has proven to be a reliable tool for screening exogenously applied compounds for their protective effects on photoreceptors [12-14]. Retinas isolated at P2 and grown in vitro for four weeks show photoreceptor degeneration comparable to that seen in vivo [15]. We and others have shown that several neurotrophic factors added in combination can significantly protect *rd1* rod photoreceptors in organ culture. Among these factors are brain-derived neurotrophic factor and glial cell line-derived neurotrophic factor, both of which are known to enhance survival and development of dopaminergic neurons in the central nervous system (CNS) [12,13]. In the vertebrate retina, dopamine (DA) plays several neuromodulatory roles, including regulation of circadian rhythms, mediation of the transition from scotopic to photopic vision, and modulation of trophic effects on retinal development and ocular growth (reviewed in [16]). DA acts through two families of G-protein coupled receptors: D1-family receptors (D1 and D5) stimulate adenylyl cyclase activity, while D2-family receptors (D2, D3, and D4) inhibit adenylyl cyclase.

We have previously shown that inhibition of DA signaling can block the degeneration of rod photoreceptors in the *rd1* retinal organ culture system for four weeks [17]. This result was achieved either through depletion of DA with 6-hydroxydopamine (6-OHDA) or with antagonists to either D1- or D2-family receptors. Replication of the protective effect of DA inhibition in vivo could lead to new therapeutic approaches for retinal degeneration. Here we have used both pharmacological and genetic approaches to determine whether the protective effects of DA inhibition can be attained in vivo in the *rd1* mouse retina.

## Methods

### Animals

Knockout (KO) mice lacking the D1, D2, D4, or D5 DA receptors (DR) were obtained from Drs. David Grandy (Vollum Institute, Oregon Health Sciences University, Portland, OR), John Drago (University of Melbourne, Parkville, VIC, Australia), and David Sibley (Molecular Neuropharmacology Section, National Institute on Neurologic Disorders and Stroke, National Institutes of Health, Bethesda, MD) [18-21]. All strains were on a congenic C57B1/6 background and were viable and fertile. Each line was crossbred with *rd1* homozygous mice, also on a C57B1/6 background, to produce heterozygous *rd1*/DR KO F1 hybrids. These mice were then sib-crossed to produce homozygous *rd1*/DR KOs for each of the four receptor subtypes. For all organ culture experiments, littermates were used from crosses of double mutants to either DR^−/−^ *rd1*/+ or DR^+/−^ *rd1*/*rd1* heterozygote mice. Genotypes were identified by PCR amplification of tail DNA with REDExtract-N-Amp PCR Kit (Sigma, St. Louis, MO) following the manufacturer’s instructions. The primers that were used are described in Table 1. All animals were handled in accordance with institutional guidelines and the National Institutes of Health Guidelines on Laboratory Animal Welfare.

**Table 1:** Primer sequences used for PCR amplification

**Gene**	**Primer (5′-3′)**
*rd1*	F: TGACAATTACTCCTTTTCCCTCAGTCTG
	R: GTAAACAGCAAGAGGCTTTATTGGGAAC
	Reverse wt primer: TACCCACCCTTCCTAATTTTTCTCACGC
D1	F: CTGATTAGCGTAGCATGGACTTTGTC
	R: TGGATGTGGAATGTGTGCGAG
	Reverse wt primer: TGGTGGCTGGAAAACATCAGA
D2	F: TGATGACTGCGAATGTTGGTGTGC
	R: AGGATTGGGAAGACAATAGCAG
	Reverse wt primer: CGGAGCCAAGCTAACACTGCAGAG
D4	F: GCCCGGTTCTTTTTGTCAAG
	Forward wt primer: CATGGACGTCATGCTGTGCA
	R: CGGACGAGTAGACCACATAG
D5	F: ACTCTCTTAATCGTCTGGACCTTG
	R: GTTCAGATCCGCCGTATCTCCTCC

### Histopathology

Histopathology was performed on four to six animals from each mutant line [15]. Histopathology reagents were purchased from EMS (Hatfield, PA). Animals were euthanized with 0.1 ml pentobarbital at P21. The eyes were enucleated, the anterior segment removed, and the remaining eyecup was fixed in 2.5% glutaraldehyde and 2% paraformaldehyde in 0.1 M phosphate buffer at 4 °C overnight, rinsed on ice two times in 0.1M phosphate buffer and two times in water for 10 min each, postfixed in 1% OsO_4_ for 1 h, rinsed two times for 10 min in water, dehydrated through a stepwise ethanol series from 50% to 100%, and embedded in Epon-Araldite. One micron plastic sections were cut on a Leica UC6 Ultramicrotome and stained with toluidine blue.

### Pharmacological studies

Intraocular (i.o.) injections were performed on wild type and *rd1* mouse pups with two injections given either at P4 and P7 or at P6 and P13. Animals were anesthetized with 75 mg ketamine/kg bodyweight and 15 mg xylazine/kg bodyweight injected intraperitoneally. Their eyelids were gently separated with forceps, and a glass micropipette connected to a Hamilton microsyringe was inserted into the vitreous and visualized through the cornea. Up to 15 μg each of 6-OHDA (Sigma) and pargyline (Sigma) in 0.5 μl sterile saline was injected. For subcutaneous (s.c.) injections, 200 mg 6-OHDA/kg bodyweight dissolved in sterile saline with 0.1% ascorbic acid was injected at the nape of the neck daily between P2–14. For combination studies, pups received s.c. injections of 300 mg 6-OHDA/kg bodyweight with 0.1% ascorbic acid daily between P2–7, except on P4, when 9 μg each of 6-OHDA and pargyline were injected i.o. Eyecups were harvested at P14 or 21 and processed either for histopathology, as described in the previous paragraph, or for tyrosine hydroxylase (TH) immunohistochemistry. For immunostaining, eyecups were fixed in 4% paraformaldehyde, rinsed in 0.1 M phosphate buffer, cryoprotected in 30% sucrose at 4 °C overnight, and frozen in Optimal Cutting Temperature Compound (EMS). Then, 10 μm cryostat sections were stained overnight at 4 °C with 1:200 goat anti-TH (Santa Cruz Biotechnology, Santa Cruz, CA) in 5% normal donkey serum, 0.3% TritonX in PBS (80 mM Na_2_HPO_4_, 20 mM NaH_2_PO_4_, 100 mM NaCl), followed by 1:200 donkey anti-goat Cy3 (Jackson ImmunoResearch, West Grove, PA) for 1 h at room temperature.

### Organ culture

Retinas from double mutant mice were grown in organ culture as previously described in detail [15]. Briefly, P2 mouse pups were anesthetized on ice, eyes were enucleated, incubated in Dulbecco’s modified Eagle’s media (DMEM; #11965; Gibco, Rockville, MD) plus 0.05% proteinase K (Invitrogen, Carlesbad CA) for 7 min at 37 °C, rinsed first in DMEM with 10% fetal calf serum (FCS; Summit Biotechnology, Ft. Collins, CO) and 1.25 μg/ml fungizone (Sigma) and then in the same media without FCS. The sclera, choroid, and anterior segment were removed using two pair of #5 forceps. The retina was separated from the retinal pigment epithelia during a 30 min incubation in DMEM with 10% FCS and 1.25 μg/ml fungizone at 37 °C. Each retina was then transferred onto a Millipore Millicell-CM culture insert (Millipore, Bedford, MA), photoreceptor side down. Cultures were incubated in DMEM with 10% FCS and 1.25 mg/ml fungizone. Cultures were maintained at 37 °C, 5% CO_2_, and fed every two to three days. Organ cultures were harvested after 27 days in vitro, fixed in mixed aldehydes, and processed for histology as described in Histopathology. Pharmacologically treated organ cultures were grown as previously described [17]. Two treatment protocols were used. Either 100 nM sulpiride (RBI Signaling, Natick, MA), the D2-family receptor antagonist, was added to the media daily immediately before feeding, or a combination of 100 μM each 6-OHDA and pargyline were added to the media on the first two days in culture with 50 μM each administered on days 7 and 8.

### Quantitative analysis

Quantitative analysis of photoreceptor survival was performed on 1 μm sections as previously described [15]. The thickness of the outer nuclear layer (ONL) was determined by an observer blinded to experimental condition. The average number of ONL cells touching each grid line on a reticule was counted in two regions on either side of the midpoint for five columns each (total of ten counts). An ANOVA statistical test was used to determine significance.

## Results

### DA depletion is incomplete in the postnatal mouse retina in vivo

We first attempted to deplete DA by adapting well established methods using 6-OHDA in the adult vertebrate retina to the neonatal *rd1* mouse [22,23]. Three approaches were taken to modify this DA depletion technique to the small eye of the postnatal mouse. First, we performed i.o. injections of 6-OHDA. Second, we injected 6-OHDA s.c. daily for 12 days beginning at P2. Finally, we combined these two methods with a single i.o. injection of 6-OHDA at P4 and s.c. injections of 6-OHDA at all other days between P2–7. Stunted growth and low survival rates were observed in animals treated with the 12 day s.c. protocol. We saw no indication of increased photoreceptor survival in retinas harvested for histology at P21 with any of the protocols (data not shown). To determine whether the DA depletion had been successful, we processed retinas for immunohistochemistry and stained them with antibodies against TH, the dopaminergic biosynthetic enzyme. In all cases examined, we observed TH-immunopositive cells in the treated retinas, indicating that the administered dose of 6-OHDA was insufficient for depletion of DA synthesis in the neonatal retina. Higher doses of 6-OHDA injected s.c. as well as intraperitoneal injections were lethal. Higher doses of 6-OHDA injected i.o. were not possible due to the small size of the postnatal eye. Thus prospects for successful DA depletion using a pharmacological approach seem poor in the postnatal in vivo mouse retina.

### DA receptor deletion does not protect *rd1* photoreceptors in vivo

**Figure 1:**
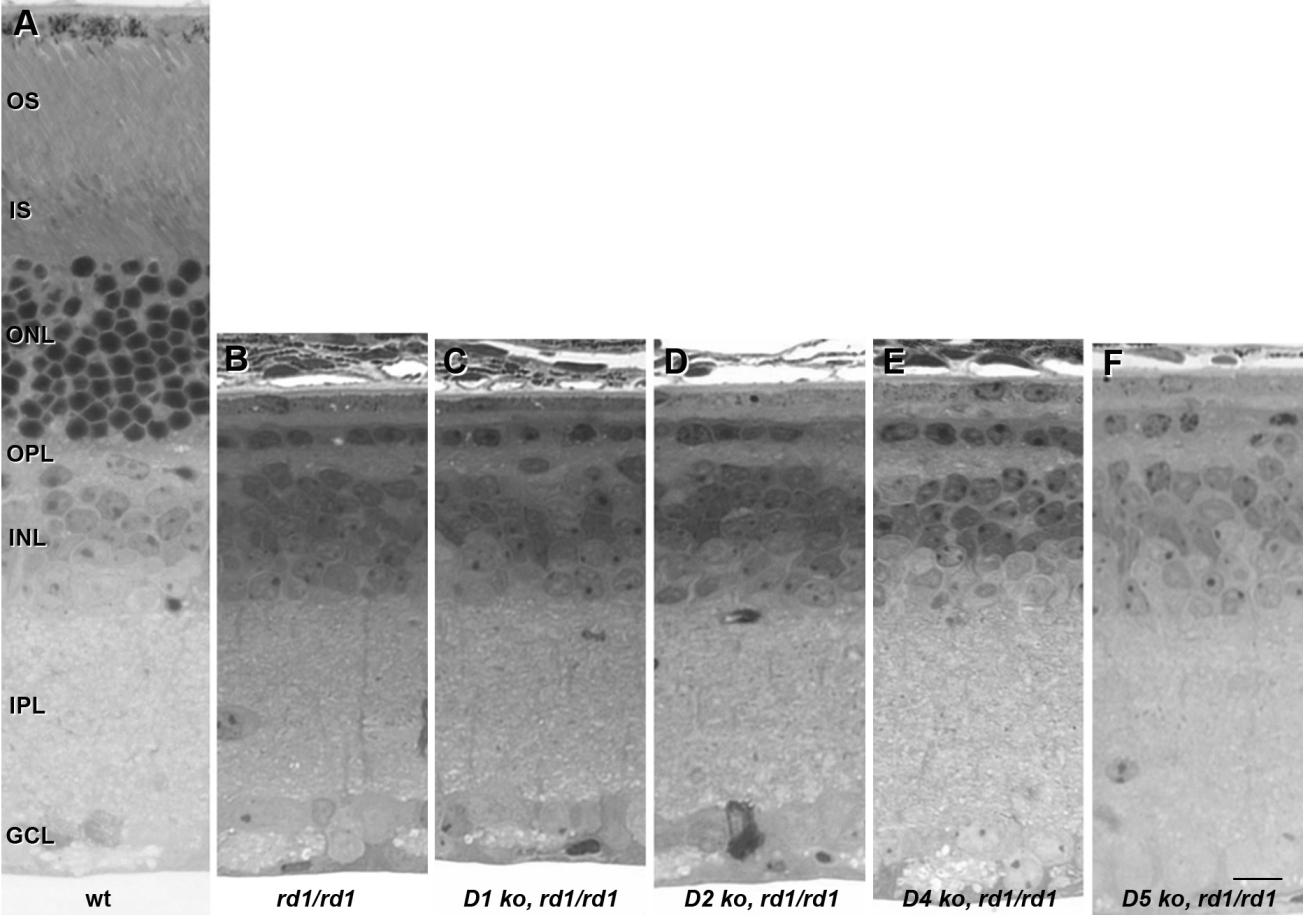
Dopamine receptor deletion does not alter photoreceptor cell survival in *rd1* retinas in vivo. Retinas from wild-type (**A**), *rd1/rd1* (**B**), *D1^−/−^, rd1/rd1* (**C**), *D2^−/−^, rd1/rd1* (**D**), *D4^−/−^, rd1/rd1* (**E**), and *D5^−/−^*, *rd1/rd1* (**F**) mice were harvested at postnatal day 21. The ONL of *rd1/rd1* retinas was reduced to a monolayer of photoreceptors, regardless of DR genotype. Abbreviations: outer segments (OS); inner segments (IS); outer nuclear layer (ONL); outer plexiform layer (OPL); inner nuclear layer (INL); inner plexiform layer (IPL); ganglion cell layer (GCL). The scale bar represents 10 μm.

We next used a genetic approach to block dopaminergic signaling in the retina. Mice deficient in each of the four DA receptor subtypes were crossed to *rd1* mice to produce animals homozygous for both mutations. These four DA receptor subtypes (D1, D2, D4, and D5) were selected for two reasons. First, they are known to be expressed in the mammalian retina [24]. Second, they are consistent with in vitro results using pharmacological antagonists that can act on each of these receptors. Eyecups were harvested at P21 for histopathology. No increase in photoreceptor survival was observed in any of the four double mutants (Figure 1). These results suggest that the protective effects of DA receptor inhibition or depletion in the organ culture cannot be replicated by deletion of a single DA receptor in vivo.

### DA receptor deletion does not protect *rd1* photoreceptors in vitro

**Figure 2:**
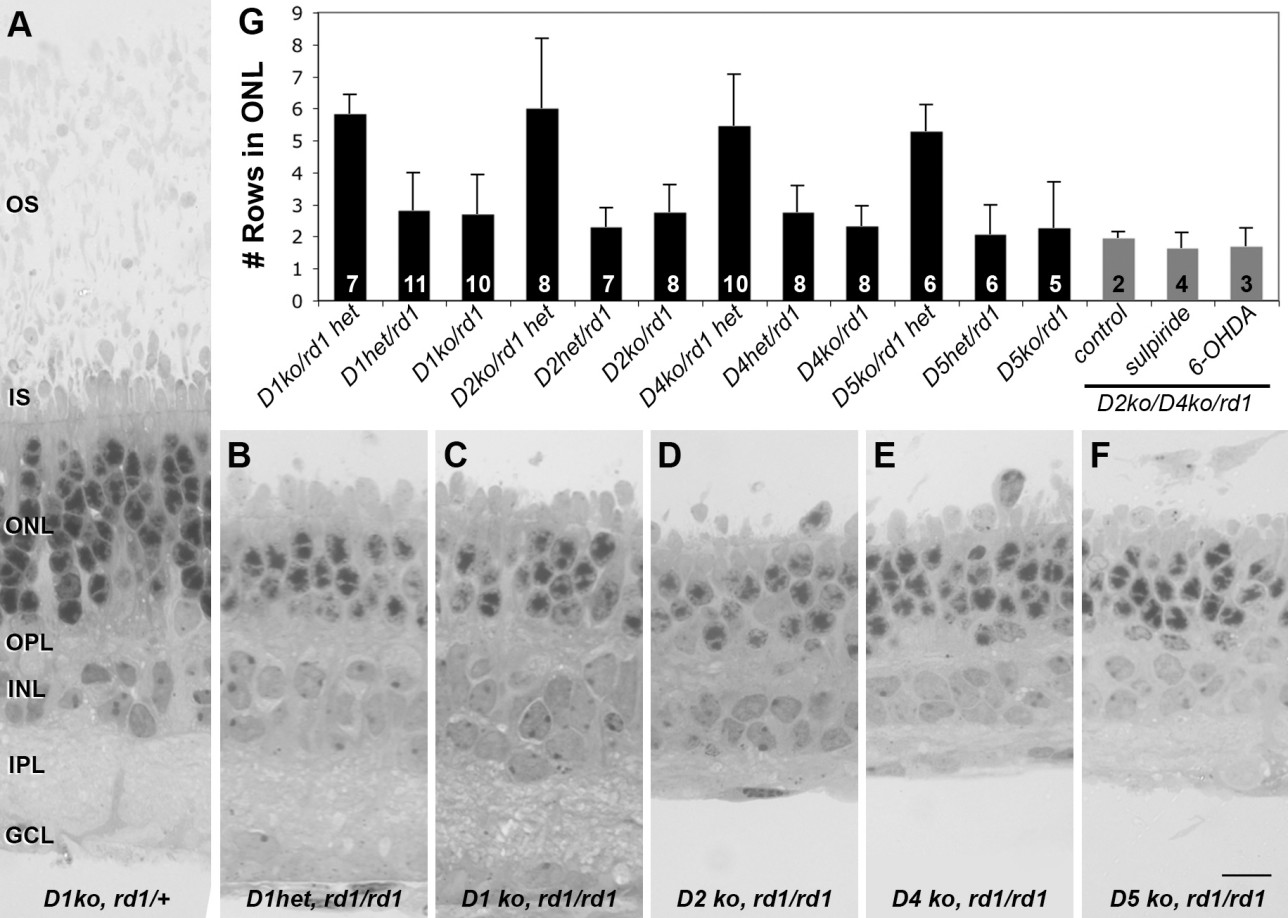
Dopamine receptor deletion does not alter photoreceptor cell survival in *rd1* retinal organ cultures. Retinas from *D1^−/−^*, *rd1/+* (**A**), *D1^+/−^*, *rd1/rd1* (**B**), *D1^−/−^*, *rd1/rd1* (**C**), *D2^−/−^*, *rd1/rd1* (**D**), *D4^−/−^*, *rd1/rd1* (**E**), and *D5^−/−^*, *rd1/rd1* (**F**) mice were harvested at postnatal day 2 and grown in organ culture for 27 days in vitro. Wild-type retinas maintained approximately five to six rows of cells in the ONL (A), while the ONL of untreated *rd1/rd1* cultures, regardless of DR genotype, was reduced to approximately two to three rows (**B**-**F**). Quantitative analysis of photoreceptor survival as measured by ONL thickness is shown in (**G**). Black bars represent the genotypes shown in **A**-**F** and additional controls. Grey bars represent organ cultures from *D2^−/−^*, *D4^−/−^, rd1/rd1* triple mutant mice treated with drugs as labeled. The number of cultures is indicated on the column for each condition; error bars indicate standard deviation. No significant difference is seen in the ONL thickness among *rd1* organ cultures, regardless of DR genetic background or treatment. Similarly, no difference is seen among rd1 heterozygous control organ cultures. Abbreviations: outer segments (OS); inner segments (IS); outer nuclear layer (ONL); outer plexiform layer (OPL); inner nuclear layer (INL); inner plexiform layer (IPL); ganglion cell layer (GCL). The scale bar represents 10 μm.

To determine whether protection of *rd1* photoreceptors by inhibition of dopaminergic signaling is a result of conditions specific to the organ culture environment, we harvested retinas from each of the double *rd1*/DR KO mutants at P2 and allowed them to grow in organ culture for 28 days. Retinas from age-matched siblings heterozygous for either the DA receptor or *rd1* were used as controls. Photoreceptor survival was not significantly increased in any of the four double mutant retinas examined (Figure 2). These results contraindicate the possibility that the increased photoreceptor survival induced by DA depletion or DA receptor inhibition is an artifact of the organ culture environment.

### Multiple DA receptor deletions do not protect *rd1* photoreceptors in vivo

**Figure 3:**
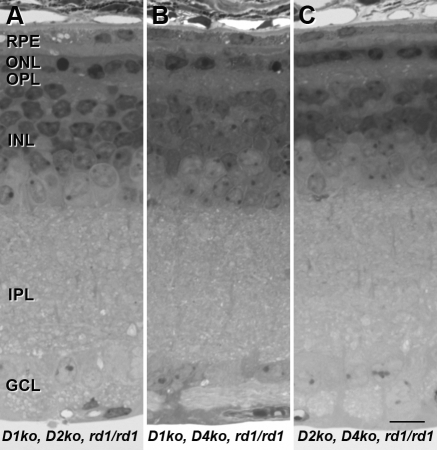
Deletion of multiple dopamine receptors does not alter photoreceptor cell survival in *rd1* retinas in vivo. Retinas from *D1^−/−^, D2^−/−^*, *rd1/rd1* (**A**), *D1^−/−^, D4^−/−^*, *rd1/rd1* (**B**), *D2^−/−^, D4^−/−^*, *rd1/rd1* (**C**) mice were harvested at postnatal day 21. The ONL of *rd1/rd1* retinas was reduced to a monolayer of photoreceptors, regardless of DR genotype. Abbreviations: retinal pigment epithelium (RPE); outer nuclear layer (ONL); outer plexiform layer (OPL); inner nuclear layer (INL); inner plexiform layer (IPL); ganglion cell layer (GCL). The scale bar represents 10 μm.

Because the protective effects of DA antagonists in organ culture could act on feedback loops involving multiple receptor subtypes, we created three triple mutants by crossing DR KO mice lacking the most abundant DA receptors in the retina, D1 and D2, with those lacking the D4 receptor, which is the only family member demonstrated to be expressed in mammalian photoreceptors [25]. D5 receptors were not included in this experiment since there is no evidence to suggest they would play a significant role independently of D1 receptors. No increased photoreceptor survival was observed at P21 in animals with the following three genotypes: (a) D1^−/−^, D2^−/−^, *rd1/rd1*; (b) D1^−/−^, D4^−/−^, *rd1/rd1*; and (c) D2^−/−^, D4^−/−^, *rd1/rd1* (Figure 3). These results suggest that the failure of the genetic approach to recapitulate the protective effects of DA depletion or DA receptor inhibition seen in *rd1* retinal organ culture is unlikely to result from redundancy of DA signaling pathways in the KO mice.

To test the possibility that an alternative signaling pathway may be responsible for the protective effects of DA inhibition previously observed in organ culture, we treated organ cultures from D2^−/−^, D4^−/−^, *rd1/rd1* triple mutant mice either with the D2-family receptor antagonist, sulpiride, or with the dopaminergic toxin, 6-OHDA. This triple mutant was selected because it includes the only DA receptor known to be expressed by photoreceptor cells and all related family members known to be expressed in the retina. Although both of these drugs produce complete protection of photoreceptors in *rd1/rd1* organ cultures, no protective effect was seen in the absence of D2-family receptors (Figure 2G), providing further evidence that DA acting through conventional dopaminergic receptors is required for photoreceptor protection.

## Discussion

DA is an important neuromodulator throughout the CNS. It is released from a very small subpopulation of interplexiform as well as amacrine neurons in the retina that have recently been demonstrated to display functional heterogeneity in both their spontaneous activity and light responses [26]. DA acts through D1- and D2-receptor families, both of which are found in the inner and outer plexiform layers [27,28]. Only the D4 receptor subtype has been localized to rod photoreceptors [25,28,29], with expression first detected around birth and peaking around P12–14 [24,30]. In the mature retina, activation of the D4 receptor supports light adaptation and inhibits the light-sensitive pool of cAMP in photoreceptors [29,31,32]. D3 receptor expression has not been detected in the vertebrate retina [24], while the role of D5 receptors is poorly understood.

In a prior study using the four-week *rd1* retinal organ culture model, we have demonstrated that photoreceptor degeneration is blocked by inhibition of DA signaling [17]. This result was achieved first through inhibition of DA receptors with either D1- or D2-family receptor antagonists (SCH-23390 and sulpiride, respectively). The finding that antagonists from both DA receptor families could block photoreceptor degeneration was a robust result. This finding is consistent with synergistic effects of D1- and D2-like receptors in locomotor control [33-35]. In contrast, D1- and D2-family receptors most often induce opposing responses in the retina [16]. Photoreceptor protection was also achieved through DA depletion with the specific dopaminergic neurotoxin, 6-OHDA. The subsequent readdition of the broad-spectrum DA agonist (±)-2-amino-6,7-dihydroxy-1,2,3,4-tetrahydronapthalene hydrobromide (ADTN) induced photoreceptor degeneration, providing powerful evidence that depletion of DA was the basis of the protection afforded by 6-OHDA and that the antagonists were acting specifically on DA receptors. Here we report that neither DA depletion with 6-OHDA nor inhibition by the D2-family receptor antagonist sulpiride provide any photoreceptor protection in organ cultures from *rd1* mice lacking both the D2 and D4 receptors. This finding provides further evidence of the specificity of the DA signaling pathway in photoreceptor protection in organ culture.

The protection observed in our prior study was striking in that photoreceptor degeneration was completely blocked as evidenced not only by statistical analysis but also confirmed by a trained observer; the observer, who was blinded to experimental condition, was unable to detect any morphological features to distinguish between wild type and 6-OHDA treated *rd1* organ cultures. In contrast, photoreceptor protection by the growth factor ciliary neurotrophic factor (CNTF) is due to negative regulation of cell differentiation, a distinction that is apparent in heterochromatin staining [36,37].

In this study, we have applied several approaches in an unsuccessful effort to reiterate the protective effects of DA depletion in the *rd1* mouse retina in vivo. Our first efforts to administer 6-OHDA through intravitreal as well as subcutaneous routes were unsuccessful due to the limiting size of the postnatal mouse eye and lethal effects of systemic inhibition of DA signaling. Consistent with our results, mice lacking both D1 and D2 receptors have been shown to survive for no more than two to three weeks, most likely due to decreased food intake and dysfunction of the gastrointestinal system [38]. Since these studies were unsuccessful for technical reasons, the underlying question of whether pharmacological inhibition of DA signaling might be protective for *rd1* photoreceptors in vivo remains unanswered.

As an alternative, we used a genetic approach by crossing mice lacking each of the four DA receptors expressed in the retina with *rd1* mice. Again, no increased photoreceptor survival was seen in vivo. Furthermore, no photoreceptor protection was observed in retinas from these double mutant mice grown in organ culture. Although the organ culture system recapitulates the photoreceptor degeneration of the *rd1* retina in vivo and has proven to be a useful tool for investigating protective compounds in the *rd1* retina, it nevertheless differs from the in vivo retina. If the protective effects of DA inhibition in the *rd1* organ culture were due to the unique characteristics of the explant system, then we would expect to see increased photoreceptor survival in organ cultures of DR KO retinas on an *rd1* background. The fact that this was not the case suggests that the protective effects of DA signaling inhibition are not simply an artifact of the organ culture system.

Finally, we considered the possibility that deletion of a single DA receptor could be insufficient to mimic the effects of DA antagonists that act on all members of a particular DA receptor family. Our previous observation that both D1- and D2-family antagonists could block photoreceptor degeneration would be consistent with an interaction among different types of DA receptors, possibly through a feedback mechanism or heterodimerization. To test this possibility, we bred triple mutant mice lacking two DA receptors (D1/D2; D1/D4; and D2/D4) on a *rd1* background. In all cases, no protective effect was observed. In light of these negative results, D5 double mutants were not tested. Since D5 receptors have no known effect on photoreceptors, additional experiments seem unlikely to produce results that would elucidate our findings. Our observations with the double mutant lines tested are consistent with a failure to detect a compensatory increase of D2-like receptors in the nucleus accumbens, caudoputamen and other striatal regions in D1 KO mice [18,39,40]. However, other compensatory effects have been detected in DA receptor deficient mice as will be discussed.

Efforts to understand why DA inhibition has such a powerful protective effect in the organ culture model, yet has not been demonstrated in vivo, is confounded by our poor understanding of the molecular mechanisms that precede photoreceptor cell death in the *rd1* retina. The *rd1* mouse retina is a well characterized animal model of autosomal recessive RP. A mutation in exon 7 of *Pde6b* results in a nonfunctional protein and leads to a tenfold increase in cytoplasmic levels of cGMP [2], an important second messenger signaling molecule in rod phototransduction. High levels of Ca^2+^ have also been detected in the *rd1* retina before degeneration, presumably due to opening of cGMP-gated cation channels [41,42].

Elevated cGMP is a feature of several models of RP in various species, including cat, dog, and mouse [43-47], and yet little is known about how cGMP induces cell death. Most of these dystrophies belong to a subset of early onset retinal degenerations. Pathology of the *rd1* retina is consistent with a failure of rod photoreceptors to undergo normal cell differentiation. Specifically, rod inner segment growth is stunted in the *rd1* retina by P6 [48,49], and the photoreceptor ribbon synapse fails to form the characteristic triad structure [50]. Interestingly, PDE is expressed in developing retina by embryonic day 12, much earlier than other genes that are required for phototransduction [51]. Rhodopsin expression, for example, is first detected in whole retina at P5, corresponding with growth of the outer segment [51]. Together, these data suggest that regulation of cGMP levels by PDE6 may play a role in photoreceptor differentiation independent of its role in phototransduction in the mature retina.

In addition, studies indicate that DA can alter cell differentiation and neurite outgrowth during development of the vertebrate retina [52-55]. D1 and D5 receptors, in particular, are expressed at high levels embryonically [24]. Similar observations have been made throughout the CNS suggesting a significant role for DA in neuronal development and differentiation [56-60]. These observations allow for the possibility that genetic deletion of DRs in KO mice could lead to modifications during embryonic retinal development that may subsequently alter postnatal pathways that are essential to the protective effects of DA inhibition observed in the postnatal organ culture.

The failure of gene inactivation to mimic the effects of antagonist drug administration has been observed in several studies involving dopaminergic pathways and can often be attributed to compensatory effects that take place during development of the KO animal [61,62]. A striking example is seen in locomotor and grooming behaviors, which are stimulated by D1 agonists and, paradoxically, enhanced in D1 receptor KO mice [63,64]. Similarly, locomotor activity is strongly reduced by D2 antagonists, but significantly less of an effect is seen in D2 receptor KO mice [65]. Interestingly, D1- and D2-like receptor agonists act in a synergistic manner to increase locomotor behavior even though they act through different signaling pathways [33-35]. These results parallel our observations that both D1- and D2-receptor antagonists are protective of photoreceptors in *rd1* retinal organ cultures, but gene deletion fails to recapitulate this protection.

Other compensatory effects in DR KO mice have been reported, such as altered levels of DA and its metabolites in the midbrain of D1R and D2R KO mice [66,67]. Further evidence supports genetic interdependence between DA receptors and other neurotransmitter receptors including adenosine A_2A_ receptors and glutamatergic N-methyl D-aspartate (NMDA) receptors [68-71]. For example, in the D4R KO mouse, increased D1R and NMDA receptor binding have been observed in both the nucleus accumbens and caudate putamen, while only NMDA receptor binding was increased in the hippocampus; no change was seen in D2R binding [71]. Similarly, A_2A_ receptor binding was increased in several brain regions in both D1R and D2R KO mice [69]. Alterations in several neuromodulatory pathways, including decreases in dynorphin and substance P expression, have been demonstrated in D1R KO mouse striatum [18,40,72]. Together, these studies and others point to the complex, pleiotrophic effects of gene inactivation and are consistent with the existence of functional interactions among these signaling systems during development. Efforts to elucidate these effects have focused on making conditional and inducible KO mice. The creation of inducible DR KO mice, with gene inactivation triggered at the same developmental age as used for the organ cultures (P2), would allow for further investigation of developmental regulation due to loss of DA receptor function during terminal photoreceptor cell differentiation.

In conclusion, the dramatic protection of *rd1* rod photoreceptors by inhibition of DA signaling in organ culture has not been reproduced in vivo by either pharmacological or genetic manipulations. Pharmacological efforts were limited by the small size of the postnatal mouse eye combined with lethal effects in the developing animal. Thus technical limitations have prevented inhibition of DA signaling in the postnatal retina, leaving the question of potential therapeutic relevance unanswered. The genetic approach, by comparison, does block DA signaling through inactivation of each DA receptor, but this approach is protective neither in vivo nor in organ culture. Further studies are needed to determine whether compensatory pathways that may alter cGMP signaling occur during development in the DR KO mouse retinas.

## References

[1] Keeler CE The Inheritance of a Retinal Abnormality in White Mice. Proc Natl Acad Sci USA 1924 10 329 33 16576828 10.1073/pnas.10.7.329 PMC1085675

[2] Farber DB Flannery JG Bowes-Rickman C The *rd* Mouse Story: Seventy Years of Research on an Animal Model of Inherited Retinal Degeneration. Prog Retin Eye Res 1994 13 31 64

[3] Bowes C Li T Dancinger M Baxter LC Applebury ML Farber DB Retinal degeneration in the *rd* mouse is caused by a defect in the β subunit of rod cGMP-phosphodiesterase. Nature 1990 347 677 80 1977087 10.1038/347677a0

[4] Lolley RN Rayborn M Hollyfield J Farber D Cyclic GMP and visual cell degeneration in the inherited disorder of rd mice: a progress report. Vision Res 1980 20 1157 61 6267810 10.1016/0042-6989(80)90054-1

[5] Pittler SJ Baehr W Identification of a nonsense mutation in the rod photoreceptor cGMP phosphodiesterase β-subunit gene of the *rd* mouse. Proc Natl Acad Sci USA 1991 88 8322 6 1656438 10.1073/pnas.88.19.8322 PMC52500

[6] Carter-Dawson LD LaVail MM Sidman RL Differential effect of the rd mutation on rods and cones in the mouse retina. Invest Ophthalmol Vis Sci 1978 17 489 98 659071

[7] Portera-Cailliau C Sung C-H Nathans J Adler R Apoptotic photoreceptor cell death in mouse models of retinitis pigmentosa. Proc Natl Acad Sci USA 1994 91 974 8 8302876 10.1073/pnas.91.3.974 PMC521436

[8] Bayes M Giordano M Balcells S Grinberg D Vilageliu L Martinez I Ayuso C Benítez J Ramos-Arroyo MA Chivelet P Solans T Valverde D Amselem S Goossens M Baiget M Gonzàlez-Duarte R Besmond C Homozygous tandem duplication within the gene encoding the beta-subunit of rod phosphodiesterase as a cause for autosomal recessive retinitis pigmentosa. Hum Mutat 1995 5 228 34 7599633 10.1002/humu.1380050307

[9] McLaughlin ME Ehrhart TL Berson EL Dryja TP Mutation spectrum of the gene encoding the β subunit of rod phosphodiesterase among patients with autosomal recessive retinitis pigmentosa. Proc Natl Acad Sci USA 1995 92 3249 53 7724547 10.1073/pnas.92.8.3249 PMC42143

[10] McLaughlin ME Sandberg MA Berson EL Dryja TP Recessive mutations in the gene encoding the β-subunit of rod phosphodiesterase in patients with retinitis pigmentosa. Nat Genet 1993 4 130 4 8394174 10.1038/ng0693-130

[11] Delyfer MN Leveillard T Mohand-Said S Hicks D Picaud S Sahel JA Inherited retinal degenerations: therapeutic prospects. Biol Cell 2004 96 261 9 15145530 10.1016/j.biolcel.2004.01.006

[12] Caffe AR Soderpalm A Holmqvist I van Veen T A combination of CNTF and BDNF rescues *rd* photoreceptors but changes rod differentiation in the presence of RPE in retinal explants. Invest Ophthalmol Vis Sci 2001 42 275 82 11133879

[13] Ogilvie JM Speck JD Lett JM Growth Factors In Combination, But Not Individually, Rescue *rd* Mouse Photoreceptors in Organ Culture. Exp Neurol 2000 161 676 85 10686086 10.1006/exnr.1999.7291

[14] Rohrer B Ogilvie JM Retarded outer segment development in TrkB knockout mouse retina organ culture. Mol Vis 2003 9 18 23 12552255

[15] Ogilvie JM Speck JD Lett JM Fleming TT A reliable method for organ culture of neonatal mouse retina with long-term survival. J Neurosci Methods 1999 87 57 65 10065994 10.1016/s0165-0270(98)00157-5

[16] Witkovsky P Dopamine and retinal function. Doc Ophthalmol 2004 108 17 40 15104164 10.1023/b:doop.0000019487.88486.0a

[17] Ogilvie JM Speck JD Dopamine Has a Critical Role in Photoreceptor Degeneration in the *rd* Mouse. Neurobiol Dis 2002 10 33 40 12079402 10.1006/nbdi.2002.0489

[18] Drago J Gerfen C Lachowicz J Steiner H Hollon T Love P Ooi GT Grinberg A Lee EJ Huang SP Bartlett PF Jose PA Sibley DR Westphal H Altered striatal function in a mutant mouse lacking D1A dopamine receptors. Proc Natl Acad Sci USA 1994 91 12564 8 7809078 10.1073/pnas.91.26.12564 PMC45479

[19] Rubinstein M Phillips TJ Bunzow JR Falzone TL Dziewczapolski G Zhang G Fang Y Larson JL McDougall JA Chester JA Saez C Pugsley TA Gershanik O Low MJ Grandy DK Mice lacking dopamine D4 receptors are supersensitive to ethanol, cocaine, and methamphetamine. Cell 1997 90 991 1001 9323127 10.1016/s0092-8674(00)80365-7

[20] Kelly MA Rubinstein M Phillips TJ Lessov CN Burkhart-Kasch S Zhang G Bunzow JR Fang Y Gerhardt GA Grandy DK Low MJ Locomotor activity in D2 dopamine receptor-deficient mice is determined by gene dosage, genetic background, and developmental adaptations. J Neurosci 1998 18 3470 9 9547254 10.1523/JNEUROSCI.18-09-03470.1998 PMC6792649

[21] Hollon TR Bek MJ Lachowicz JE Ariano MA Mezey E Ramachandran R Wersinger SR Soares-da-Silva P Liu ZF Grinberg A Drago J Young WS 3rd Westphal H Jose PA Sibley DR Mice lacking D5 dopamine receptors have increased sympathetic tone and are hypertensive. J Neurosci 2002 22 10801 10 12486173 10.1523/JNEUROSCI.22-24-10801.2002 PMC6758465

[22] Ehinger B Nordenfelt L Destruction of retinal dopamine-containing neurons in rabbit and goldfish. Exp Eye Res 1977 24 179 87 844511 10.1016/0014-4835(77)90258-5

[23] Lin Z-S Yazulla S Depletion of retinal dopamine increases brightness perception in goldfish. Vis Neurosci 1994 11 683 93 7918219 10.1017/s0952523800002996

[24] Fujieda H Scher J Lukita-Atmadja W Brown GM Gene regulation of melatonin and dopamine receptors during eye development. Neuroscience 2003 120 301 7 12890503 10.1016/s0306-4522(03)00298-7

[25] Cohen AI Todd RD Harmon S O'Malley KL Photoreceptors of mouse retinas possess D4 receptors coupled to adenylate cyclase. Proc Natl Acad Sci USA 1992 89 12093 7 1334557 10.1073/pnas.89.24.12093 PMC50704

[26] Zhang DQ Zhou TR McMahon DG Functional heterogeneity of retinal dopaminergic neurons underlying their multiple roles in vision. J Neurosci 2007 27 692 9 17234601 10.1523/JNEUROSCI.4478-06.2007 PMC6672798

[27] Witkovsky P Schütte M The organization of dopaminergic neurons in vertebrate retinas. Vis Neurosci 1991 7 113 24 1931794 10.1017/s0952523800010981

[28] Nguyen-Legros J Versaux-Botteri C Vernier P Dopamine receptor localization in the mammalian retina. Mol Neurobiol 1999 19 181 204 10495103 10.1007/BF02821713

[29] Nir I Harrison JM Haque R Low MJ Grandy DK Rubinstein M Iuvone PM Dysfunctional light-evoked regulation of cAMP in photoreceptors and abnormal retinal adaptation in mice lacking dopamine D4 receptors. J Neurosci 2002 22 2063 73 11896146 10.1523/JNEUROSCI.22-06-02063.2002 PMC6758276

[30] Klitten LL Rath MF Coon SL Kim JS Klein DC Moller M Localization and regulation of dopamine receptor D4 expression in the adult and developing rat retina. Exp Eye Res 2008 87 471 7 18778704 10.1016/j.exer.2008.08.004 PMC2597030

[31] Cohen AI Blazynski C Dopamine and its agonists reduce a light-sensitive pool of cyclic AMP in mouse photoreceptors. Vis Neurosci 1990 4 43 52 1702315 10.1017/s0952523800002753

[32] Jackson CR Chaurasia SS Zhou H Haque R Storm DR Iuvone PM Essential roles of dopamine D4 receptors and the type 1 adenylyl cyclase in photic control of cyclic AMP in photoreceptor cells. J Neurochem 2009 109 148 57 19166506 10.1111/j.1471-4159.2009.05920.x PMC2727872

[33] Daly SA Waddington JL Two directions of dopamine D1/D2 receptor interaction in studies of behavioural regulation: a finding generic to four new, selective dopamine D1 receptor antagonists. Eur J Pharmacol 1992 213 251 8 1387846 10.1016/0014-2999(92)90689-2

[34] Waddington JL Daly SA Downes RP Deveney AM McCauley PG O'Boyle KM Behavioural pharmacology of 'D-1-like' dopamine receptors: further subtyping, new pharmacological probes and interactions with 'D-2-like' receptors. Prog Neuropsychopharmacol Biol Psychiatry 1995 19 811 31 8539421 10.1016/0278-5846(95)00130-n

[35] White FJ Bednarz LM Wachtel SR Hjorth S Brooderson RJ Is stimulation of both D1 and D2 receptors necessary for the expression of dopamine-mediated behaviors? Pharmacol Biochem Behav 1988 30 189 93 2902644 10.1016/0091-3057(88)90442-x

[36] Schulz-Key S Hofmann H Beisenherz-Huss C Barbisch C Kirsch M Ciliary neurotrophic factor as a transient negative regulator of rod development in rat retina. Invest Ophthalmol Vis Sci 2002 43 3099 108 12202535

[37] Ogilvie JM Photoreceptor Rescue in an Organotypic Model of Retinal Degeneration. Prog Brain Res 2001 131 641 8 11420977 10.1016/s0079-6123(01)31050-6

[38] Kobayashi M Iaccarino C Saiardi A Heidt V Bozzi Y Picetti R Vitale C Westphal H Drago J Borrelli E Simultaneous absence of dopamine D1 and D2 receptor-mediated signaling is lethal in mice. Proc Natl Acad Sci USA 2004 101 11465 70 15272078 10.1073/pnas.0402028101 PMC509223

[39] Xu M Hu XT Cooper DC Moratalla R Graybiel AM White FJ Tonegawa S Elimination of cocaine-induced hyperactivity and dopamine-mediated neurophysiological effects in dopamine D1 receptor mutant mice. Cell 1994 79 945 55 8001143 10.1016/0092-8674(94)90026-4

[40] Xu M Moratalla R Gold LH Hiroi N Koob GF Graybiel AM Tonegawa S Dopamine D1 receptor mutant mice are deficient in striatal expression of dynorphin and in dopamine-mediated behavioral responses. Cell 1994 79 729 42 7954836 10.1016/0092-8674(94)90557-6

[41] Fox DA Poblenz AT He L Harris JB Medrano CJ Pharmacological strategies to block rod photoreceptor apoptosis caused by calcium overload: a mechanistic target-site approach to neuroprotection. Eur J Ophthalmol 2003 13 Suppl 3 S44 56 12749677 10.1177/112067210301303s08

[42] Fox DA Poblenz AT He L Calcium overload triggers rod photoreceptor apoptotic cell death in chemical-induced and inherited retinal degenerations. Ann N Y Acad Sci 1999 893 282 5 10672249 10.1111/j.1749-6632.1999.tb07837.x

[43] Kommonen B Kylma T Cohen R Penn J Paulin L Hurwitz M Hurwitz RL Elevation of cGMP with normal expression and activity of rod cGMP-PDE in photoreceptor degenerate labrador retrievers. Ophthalmic Res 1996 28 19 28 8726673 10.1159/000267869

[44] Voaden MJ Curtis R Barnett K Leon A Doshi M Hussain A Dominant rod-cone dysplasia in the Abyssinian cat. Prog Clin Biol Res 1987 247 369 80 2825215

[45] Woodford BJ Liu Y Fletcher R Chader G Farber D Santos-Anderson R Tso MO Cyclic nucleotide metabolism in inherited retinopathy in collies: a biochemical and histochemical study. Exp Eye Res 1982 34 703 14 6282610 10.1016/s0014-4835(82)80031-6

[46] Aquirre G Farber D Lolley R Fletcher R Chader G Rod-cone dysplasia in Irish setters: a defect in cyclic GMP metabolism in visual cells. Science 1978 201 1133 4 210508 10.1126/science.210508

[47] Chang B Hawes NL Hurd RE Davisson MT Nusinowitz S Heckenlively JR Retinal degeneration mutants in the mouse. Vision Res 2002 42 517 25 11853768 10.1016/s0042-6989(01)00146-8

[48] Caley DW Johnson C Liebelt RA The Postnatal Development of the Retina in the Normal and Rodless CBA Mouse: A Light and Electron Microscopic Study. Am J Anat 1972 133 179 211 5009246 10.1002/aja.1001330205

[49] Sanyal S Bal AK Comparative Light and Electron Microscopic Study of Retinal Histogenesis in Normal and *rd* Mutant Mice. Z Anat Entwicklungsgesch 1973 142 219 38 4781863 10.1007/BF00519723

[50] Blanks JC Adinolfi AM Lolley RN Photoreceptor Degeneration and Synaptogenesis in Retinal-degenerative (rd) Mice. J Comp Neurol 1974 156 95 106 4836657 10.1002/cne.901560108

[51] Bibb LC Holt JK Tarttelin EE Hodges MD Gregory-Evans K Rutherford A Lucas RJ Sowden JC Gregory-Evans CY Temporal and spatial expression patterns of the CRX transcription factor and its downstream targets. Critical differences during human and mouse eye development. Hum Mol Genet 2001 10 1571 9 11468275 10.1093/hmg/10.15.1571

[52] Lankford K De Mello FG Klein WL A transient embryonic dopamine receptor inhibits growth cone motility and neurite outgrowth in a subset of avian retina neurons. Neurosci Lett 1987 75 169 74 2952906 10.1016/0304-3940(87)90292-8

[53] Lankford KL DeMello FG Klein WL D1-type dopamine receptors inhibit growth cone motility in cultured retina neurons: evidence that neurotransmitters act as morphogenic growth regulators in the developing central nervous system. Proc Natl Acad Sci USA 1988 85 4567 71 3380807 10.1073/pnas.85.12.4567-a PMC280472

[54] Rodrigues Pdos S Dowling JE Dopamine induces neurite retraction in retinal horizontal cells via diacylglycerol and protein kinase C. Proc Natl Acad Sci USA 1990 87 9693 7 2263620 10.1073/pnas.87.24.9693 PMC55239

[55] Guimaraes MZ Hokoc JN Duvoisin R Reis RA De Mello FG Dopaminergic retinal cell differentiation in culture: modulation by forskolin and dopamine. Eur J Neurosci 2001 13 1931 7 11403686 10.1046/j.0953-816x.2001.01575.x

[56] Lauder JM Neurotransmitters as growth regulatory signals: role of receptors and second messengers. Trends Neurosci 1993 16 233 40 7688165 10.1016/0166-2236(93)90162-f

[57] Levitt P Harvey JA Friedman E Simansky K Murphy EH New evidence for neurotransmitter influences on brain development. Trends Neurosci 1997 20 269 74 9185309 10.1016/s0166-2236(96)01028-4

[58] Nguyen L Rigo JM Rocher V Belachew S Malgrange B Rogister B Leprince P Moonen G Neurotransmitters as early signals for central nervous system development. Cell Tissue Res 2001 305 187 202 11545256 10.1007/s004410000343

[59] McCarthy D Lueras P Bhide PG Elevated dopamine levels during gestation produce region-specific decreases in neurogenesis and subtle deficits in neuronal numbers. Brain Res 2007 1182 11 25 17950709 10.1016/j.brainres.2007.08.088 PMC2141544

[60] Ohtani N Goto T Waeber C Bhide PG Dopamine modulates cell cycle in the lateral ganglionic eminence. J Neurosci 2003 23 2840 50 12684471 10.1523/JNEUROSCI.23-07-02840.2003 PMC1201391

[61] Gingrich JA Hen R The broken mouse: the role of development, plasticity and environment in the interpretation of phenotypic changes in knockout mice. Curr Opin Neurobiol 2000 10 146 52 10679442 10.1016/s0959-4388(99)00061-6

[62] Waddington JL O'Tuathaigh C O'Sullivan G Tomiyama K Koshikawa N Croke DT Phenotypic studies on dopamine receptor subtype and associated signal transduction mutants: insights and challenges from 10 years at the psychopharmacology-molecular biology interface. Psychopharmacology (Berl) 2005 181 611 38 16041535 10.1007/s00213-005-0058-8

[63] Clifford JJ Tighe O Croke DT Kinsella A Sibley DR Drago J Waddington JL Conservation of behavioural topography to dopamine D1-like receptor agonists in mutant mice lacking the D1A receptor implicates a D1-like receptor not coupled to adenylyl cyclase. Neuroscience 1999 93 1483 9 10501473 10.1016/s0306-4522(99)00297-3

[64] Clifford JJ Tighe O Croke DT Sibley DR Drago J Waddington JL Topographical evaluation of the phenotype of spontaneous behaviour in mice with targeted gene deletion of the D1A dopamine receptor: paradoxical elevation of grooming syntax. Neuropharmacology 1998 37 1595 602 9886682 10.1016/s0028-3908(98)00116-6

[65] Kelly MA Rubinstein M Phillips TJ Lessov CN Burkhart-Kasch S Zhang G Bunzow JR Fang Y Gerhardt GA Grandy DK Low MJ Locomotor activity in D2 dopamine receptor-deficient mice is determined by gene dosage, genetic background, and developmental adaptations. J Neurosci 1998 18 3470 9 9547254 10.1523/JNEUROSCI.18-09-03470.1998 PMC6792649

[66] El-Ghundi M George SR Drago J Fletcher PJ Fan T Nguyen T Liu C Sibley DR Westphal H O'Dowd BF Disruption of dopamine D1 receptor gene expression attenuates alcohol-seeking behavior. Eur J Pharmacol 1998 353 149 58 9726645 10.1016/s0014-2999(98)00414-2

[67] Parish CL Finkelstein DI Drago J Borrelli E Horne MK The role of dopamine receptors in regulating the size of axonal arbors. J Neurosci 2001 21 5147 57 11438590 10.1523/JNEUROSCI.21-14-05147.2001 PMC6762846

[68] Dassesse D Massie A Ferrari R Ledent C Parmentier M Arckens L Zoli M Schiffmann SN Functional striatal hypodopaminergic activity in mice lacking adenosine A(2A) receptors. J Neurochem 2001 78 183 98 11432985 10.1046/j.1471-4159.2001.00389.x

[69] Short JL Ledent C Borrelli E Drago J Lawrence AJ Genetic interdependence of adenosine and dopamine receptors: evidence from receptor knockout mice. Neuroscience 2006 139 661 70 16476524 10.1016/j.neuroscience.2005.12.052

[70] Holmes A Lachowicz JE Sibley DR Phenotypic analysis of dopamine receptor knockout mice; recent insights into the functional specificity of dopamine receptor subtypes. Neuropharmacology 2004 47 1117 34 15567422 10.1016/j.neuropharm.2004.07.034

[71] Gan L Falzone TL Zhang K Rubinstein M Baldessarini RJ Tarazi FI Enhanced expression of dopamine D(1) and glutamate NMDA receptors in dopamine D(4) receptor knockout mice. J Mol Neurosci 2004 22 167 78 14997010 10.1385/JMN:22:3:167

[72] Wong JY Clifford JJ Massalas JS Finkelstein DI Horne MK Waddington JL Drago J Neurochemical changes in dopamine D1, D3 and D1/D3 receptor knockout mice. Eur J Pharmacol 2003 472 39 47 12860471 10.1016/s0014-2999(03)01862-4

